# Audit of Benzodiazepine and Z-Drugs Prescription in an Acute Inpatient Adult Psychiatric Unit in North Wales With the Aim of a Quality Improvement Project

**DOI:** 10.1192/bjo.2025.10571

**Published:** 2025-06-20

**Authors:** Ashna Anil, Simran Bhupal, Sathyan Soundararajan, Chloe Turner, Asha Dhandapani

**Affiliations:** 1 BCUHB, Wrexham, United Kingdom; 2 BCUHB, Bangor, United Kingdom

## Abstract

**Aims:** This audit aims to assess the prescribing practices of benzodiazepines and hypnotics within the Heddfan inpatient psychiatric unit. These medications are effective in managing acute anxiety, agitation, and insomnia; however, they carry significant risks, including dependency, cognitive impairment, and an increased likelihood of falls.

This audit seeks to identify prescribing patterns, evaluate compliance with established guidelines, and suggest recommendations to enhance clinical usage, in light of the significant prevalence of their use and misuse as indicated by recent data.

The aims are as follows:

1. Evaluate the prevalence and patterns of benzodiazepine and hypnotic prescribing in psychiatric inpatient settings.

2. Assess adherence to national guidelines, including treatment duration and dosage limits.

3. Identify factors contributing to misuse or overprescribing, such as prolonged PRN use or multiple concomitant prescriptions.

4. Provide evidence-based recommendations to enhance patient safety and prescribing practices.

**Methods:** The audit will involve a retrospective review of medical records over a month in the Heddfan Psychiatric Unit. Data will be collected and analysed to evaluate prescribing patterns, including:

Frequency of benzodiazepine and hypnotic prescriptions.

Duration and dosages of treatment.

Documentation of prescribing rationale and adherence to guidelines.

Data Collection: The following data points were collected:

Patient demographics (age, gender, diagnosis, and length of stay).

Type of benzodiazepine or hypnotic prescribed (e.g. lorazepam, zolpidem).

Dosage and duration of each prescription.

Indication for use (e.g. agitation, insomnia, anxiety).

Use of multiple benzodiazepines or hypnotics simultaneously.

Adverse events reported during treatment (e.g. falls, agitation).

Discharge plans, including tapering schedules or withdrawal strategies.

**Results:** Of the total sample observed, out of the 40 patients, 38 were prescribed PRN benzodiazepines. The indication was agitation in about 18 and anxiety in the other 20.

Most patients studied were commenced on lorazepam (35 of 38; 92%) while the rest received diazepam (3 of 38).

Out of the 38 patients, only 12 were reviewed for PRN medication in a week. Only one patient had their PRN medicines stopped while exactly half continued receiving PRN benzodiazepine for more than 3 weeks. 28 patients out of 38 were written up for a maximum dose of up to 4 mg per day. 2 patients received 8 mg and one patient received 15 mg diazepam.

50% (20) of patients received zopiclone as a secondary sedative of which 66% (8) continued for 3 weeks or more than 3 weeks.

Of the total 20 patients, 3 (15%) were on regular benzodiazepine of which 2 received diazepam and 1 received zopiclone.

The most common concurrent antidepressant prescribed was sertraline, closely followed by mirtazapine. Among antipsychotics, there was an equal prescription of regular olanzapine, quetiapine and aripiprazole.

Of the total sample, 27.5% (11 patients) were already on regular benzodiazepine, of which 4 were receiving diazepam, followed by 3 receiving temazepam and 1 was on clonazepam.

**Conclusion:** Our recommendations from this audit are as follows:

Enhanced training for prescribers on guideline adherence.

Increased involvement of pharmacists in monitoring and auditing prescriptions.

Development of standardized protocols for prescribing and tapering benzodiazepines and hypnotics.

Promotion of non-pharmacological alternatives to manage agitation and insomnia.

We will be liaising with the pharmacist to generate a protocol for weekly reviews during ward rounds and to make a protocol for reducing PRN medications and stopping them, both while in the inpatient unit and at discharge.

